# Angiotensin II Protects Primary Rat Hepatocytes against Bile Salt-Induced Apoptosis

**DOI:** 10.1371/journal.pone.0052647

**Published:** 2012-12-26

**Authors:** Golnar Karimian, Manon Buist-Homan, Bojana Mikus, Robert H. Henning, Klaas Nico Faber, Han Moshage

**Affiliations:** 1 Department of Gastroenterology and Hepatology, University Medical Center Groningen, University of Groningen, Groningen, The Netherlands; 2 Department of Clinical Pharmacology, University Medical Center Groningen, University of Groningen, Groningen, The Netherlands; Université Paris Sud, France

## Abstract

Angiotensin II (AT-II) is a pro-fibrotic compound that acts via membrane-bound receptors (AT-1R/AT-2R) and thereby activates hepatic stellate cells (HSCs). AT-II receptor blockers (ARBs) are thus important candidates in the treatment of liver fibrosis. However, multiple case reports suggest that AT-1R blockers may induce hepatocyte injury. Therefore, we investigated the effect of AT-II and its receptor blockers on cytokine-, oxidative stress- and bile salt-induced cell death in hepatocytes. Primary rat hepatocytes were exposed to TNF-α/Actinomycin D, the ROS-generating agent menadione or the bile salts: glycochenodeoxycholic acid (GCDCA) and tauro-lithocholic acid-3 sulfate (TLCS), to induce apoptosis. AT-II (100 nmol/L) was added 10 minutes prior to the cell death-inducing agent. AT-1R antagonists (Sartans) and the AT-2R antagonist PD123319 were used at 1 µmol/L. Apoptosis (caspase-3 activity, acridine orange staining) and necrosis (Sytox green staining) were quantified. Expression of CHOP (marker for ER stress) and AT-II receptor mRNAs were quantified by Q-PCR. AT-II dose-dependently reduced GCDCA-induced apoptosis of hepatocytes (−50%, p<0.05) without inducing necrosis. In addition, AT-II reduced TLCS-induced apoptosis of hepatocytes (−50%, p<0.05). However, AT-II did not suppress TNF/Act-D and menadione-induced apoptosis. Only the AT-1R antagonists abolished the protective effect of AT-II against GCDCA-induced apoptosis. AT-II increased phosphorylation of ERK and a significant reversal of the protective effect of AT-II was observed when signaling kinases, including ERK, were inhibited. Moreover, AT-II prevented the GCDCA-induced expression of CHOP (the marker of the ER-mediated apoptosis).

**Conclusion:**

Angiotensin II protects hepatocytes from bile salt-induced apoptosis through a combined activation of PI3-kinase, MAPKs, PKC pathways and inhibition of bile salt-induced ER stress. Our results suggest a mechanism for the observed hepatocyte-toxicity of Sartans (angiotensin receptor blockers, ARBs) in some patients with chronic liver injury.

## Introduction

Angiotensin II (AT-II) is the effector peptide of the renin angiotensin system (RAS), which plays a crucial role in regulating blood pressure. In addition to the systemic generation of AT-II in the circulation by RAS, AT-II is also produced locally in various organs, including kidney, vessels, heart, adrenal gland, brain and liver. A process commonly termed as “tissue” renin-angiotensin system (RAS) mediates the local production of AT-II [1]. Tissue RAS plays an important role in maintaining cardiovascular homeostasis and in mediating diverse physiologic functions such as cell growth, cell differentiation and apoptosis [2]. The AT-II type 1 and type 2 receptors (AT-1R and AT-2R) mediate the effects of AT-II on organs [2]. It has been shown that components of the RAS are present and activated in chronic liver diseases [3], [4].

Chronic liver diseases, including cholestatic liver disease, are characterized by loss of functional liver mass due to hepatocyte cell death and the development of liver fibrosis that may progress to end-stage liver cirrhosis. Hepatic RAS is suggested to play an important role in liver fibrosis [5]. Most of the key components of RAS that lead to the generation of AT-II are present in the liver [5], [6] and are induced or redistributed in liver injury [3], [4], [7], [8]. AT-II levels are increased both in plasma and in liver tissue in rat models of liver disease, as well as in cirrhotic patients [3], [9]. It was shown that AT-II, generated by systemic RAS and/or tissue RAS, plays a role in the progression of liver fibrosis through activation and proliferation of hepatic stellate cells (HSCs) [10], [11]. Moreover, activated hepatic stellate cells express RAS-components and synthesize AT-II themselves. Although hepatocytes are the major source for angiotensinogen (the AT-II precursor) but they express less renin and angiotensin converting enzyme (ACE) than HSC [4]. Both HSC-derived AT-II and systemic AT-II can exert paracrine and endocrine actions on hepatocytes, which express high levels of AT-1R [4]. Recent studies revealed that blocking the RAS pathway with either AT-1R blockers (ARB) or ACE inhibitors (ACEi) attenuates the progression of liver fibrosis in animal models of chronic liver diseases [5], [12], [13]. Consequently, blockade of AT-II signal transduction may be a beneficial therapy in patients with chronic liver diseases. Until now, only a small number of studies examining the effect of RAS inhibition on fibrosis in human liver diseases are available and there are no results from large randomized trials (reviewed in [5]). Notably, a recent cohort study in chronic hepatitis C patients with advanced liver fibrosis showed that ACEi/ARB therapy does not prevent the progression of hepatic fibrosis [14]. On the other hand, there are multiple (case) reports indicating that ARBs and ACEis may induce hepatocellular injury and/or cholestasis [15]–[24]. Losartan and candesartan were found to induce hepatocellular injury in hypertensive patients with normal liver function tests prior to the start of the therapy [15]–[20]. Irbesartan therapy leads to hepatocyte cholestasis and degeneration in hypertensive patients [21], [22] and valsartan has been reported to induce lobular necrosis and inflammation in the liver [23], [24]. There are also numerous reports of the potential hepatotoxicity of ACE inhibitors (reviewed in [25]). Thus, inhibition of the RAS system in fibrotic liver disease may have negative effects on liver function and hepatocyte viability in particular.

In liver diseases, hepatocyte injury may be caused by (a combination of) inflammatory cytokines, oxidative stress and increased bile salt levels, leading to apoptosis and/or necrosis of hepatocytes. Therefore, we studied the effect of AT-II on cytokine-, ROS- and bile salt-induced apoptosis and necrosis in primary rat hepatocytes. AT-II specifically attenuated bile salt-induced apoptosis, but not cytokine- or oxidative stress-induced apoptosis. Subsequently, we analyzed the involvement of the AT-II receptors, protein kinase signaling pathways and ER stress in the protective effects of AT-II against bile salt-induced apoptosis in detail.

## Materials and Methods

### Animals

Specified pathogen-free male Wistar rats (220–250 g) were purchased from Charles River Laboratories Inc. (Wilmington, MA, USA). Animals were kept under standard laboratory conditions with free access to standard laboratory chow and water. The local Committee for Care and Use of laboratory animals in University of Groningen specifically approved the experiments of this study and all the experiments were performed in accordance with the guidelines of this Committee.

### Rat Hepatocyte Isolation

Hepatocytes were isolated by a two-step perfusion method using collagenase as described before [26]. In brief, the liver was first perfused through portal vein with Ca^2+^- free Krebs Ringer Hepes buffer, pH 7.4, maintained at 37°C (10 min, flow rate = 25 ml/min), followed by perfusion with Mg^2+^-free Krebs Ringer Hepes buffer containing Ca^2+^ (5.7 mmol/L) and Collagenase type I (Sigma-Aldrich; 0.12–0.16 U/ml, 10 min, flow rate 8 ml/min). The liver was then removed and placed in the same buffer containing 1% bovine serum albumin (BSA; Sigma-Aldrich) without Collagenase. Hepatocytes were then released by gentle teasing of the softened liver and filtered through 60-mesh sterile nylon gauze. The dissected inferior vena cava was used as the outflow port. The buffers were oxygenated prior to perfusion.

Cells were washed three times with HBSS at 50 g for 5 min and the supernatant was discarded. The final cell pellet was re-suspended and cultured in William’s E medium in a humidified incubator at 37°C and 5% CO_2_ as described before [27].

### Experimental Design

Experiments were started after the attachment period of 4 hours. Monolayers of cultured primary hepatocytes were treated with angiotensin II (Sigma-Aldrich) at the indicated concentrations starting 10 minutes prior to the exposure to apoptotic stimuli as follows: 50 µmol/L GCDCA (Sigma-Aldrich) for 4 hours, 100 µmol/L of TLCS (Sigma-Aldrich) for 6 hours, 20 ng/ml recombinant murine TNFα (R&D Systems, Abingdon, United Kingdom) for 16 hours, and 50 µmol/L menadione (superoxide anion donor; Sigma-Aldrich) for 9 hours unless stated otherwise. Signal transduction pathways were inhibited using 10 µmol/L of the ERK1/2 inhibitor U0126 (Promega, Madison, USA), 10 µmol/L of the p38 inhibitor SB 203580 (Calbiochem), 50 µmol/L of the PI3 kinase inhibitor LY 294002 (Calbiochem), 1 µmol/L of the protein kinase-C inhibitors Calphostin-C and Bisindolylmaleimide I (BSM-I) (Calbiochem), and 200 ng/ml of the transcriptional inhibitor actinomycin-D (Roche Diagnostics, Almere, the Netherlands). Angiotensin II type 1 receptor was inhibited by the receptor antagonists Losartan (Merck), Valsartan (Novartis Pharma), Irbesartan (Bristol-Myers-Suibb) and Candesartan (Astra Zeneca, Zoetermeer, the Netherlands) all used at 1 µmol/L. Angiotensin II type 2 receptor was inhibited by 1 µmol/L of the selective antagonist PD 123319 (Sigma-Aldrich). All inhibitors and receptor antagonists were added to the cultured hepatocytes 30 minutes prior to the apoptotic stimuli unless stated otherwise. Every experimental condition was performed in triplicate wells and each experiment was repeated at least three times using hepatocytes from different rats. Cells were harvested at the indicated time points as described previously [27].

### Apoptosis and Necrosis Assays

Caspase-3 activity was measured as described previously [27]. The arbitrary fluorescence unit (AFU) was corrected for the amount of protein. Protein concentration was determined using the Bio-Rad protein assay kit. Sytox green (Invitrogen) and acridine orange (Sigma-Aldrich) were used to visualize necrotic and apoptotic cell death, respectively, as described before [28].

### Quantitative PCR

RNA isolation, reverse transcription PCR and quantitative PCR (qPCR) were performed as described previously [28]. Each sample was analyzed in duplicate. 18S mRNA levels were used as endogenous control. Primers and probes are listed in Table S1 (Supporting information).

### Western-blot Analysis

Western blot analysis was performed as described previously [28]. Expression of selected protein was assessed using monoclonal mouse antibody against phosphorylated ERK1/2 (p44/42) at a dilution of 1∶1000. Blots were subsequently stripped using 0.1% SDS/0.1% Tween – PBS at 65°C for 30 minutes and incubated with 1∶4000 diluted monoclonal mouse antibody against GAPDH (Calbiochem, La Jolla, CA. USA). Horse radish-peroxidase conjugated rabbit anti-mouse Ig (DAKO, Denmark) was used every time as a secondary antibody at a dilution of 1∶2000.

### Statistical Analysis

Results are presented as the mean of at least 3 independent experiments ± SD. An unpaired student t-test or ANOVA test was used to determine the significance of differences between experimental groups. A P-value of less than 0.05 (P<0.05) was considered statistically significant.

## Results

### Angiotensin II Inhibits Bile Acid-induced Caspase-3 Activity and Apoptotic Nuclear Morphology

**Figure 1:**
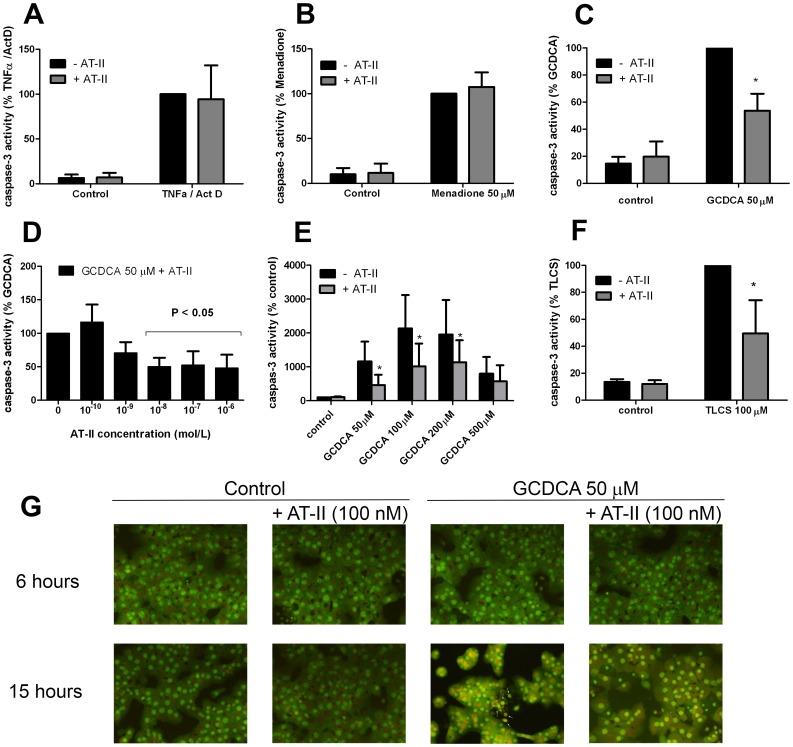
Angiotensin II (AT-II) inhibits glycochenodeoxycholic acid (GCDCA)-induced apoptosis but not TNFα- and menadione-induced apoptosis. (**a**) Caspase-3 activity in rat hepatocytes treated with 20 ng/ml of TNFα and 200 ng/ml of transcriptional inhibitor actinomycin-D for 16 hours in the presence and absence of 100 nmol/L AT- II. (**b**) Caspase-3 activity in rat hepatocytes treated with 50 µmol/L of menadione for 9 hours in the presence and absence of 100 nmol/L AT-II. (**c**) Primary rat hepatocytes were treated for 4 hours with 50 µmol/L of GCDCA, 100 nmol/L of AT-II or a combination of both. AT-II was added 10 min prior to the addition of GCDCA. Two-way ANOVA test was used to determine the significance of differences between experimental groups (N = 5). * P<0.05 for GCDCA +AT-II vs. GCDCA alone. (**d**) Hepatocytes were treated with different concentrations of AT-II 10 min prior to the addition of 50 µmol/L of GCDCA for 4 hours. One-way ANOVA test was used to determine the significance of differences between experimental groups (N = 5). (**e**) AT-II (100 nmol/L) significantly inhibits GCDCA-induced caspase-3 activation. Two-way ANOVA test was used to determine the significance of differences between experimental groups (N = 5). * P<0.05 for GCDCA (50, 100, 200 µM)+AT-II vs GCDCA (50, 100, 200 µM) alone. (**f**) AT-II inhibits tauro-lithocholic acid-3 sulfate (TLCS)-induced caspase-3 activity. Hepatocytes were treated for 6 hours with 100 µmol/L of TLCS, 100 nmol/L of AT-II or a combination of both. Cells were treated with AT-II 10 min prior to the addition of TLCS. Two-way ANOVA test was used to determine the significance of differences between experimental groups (N = 5).* P<0.05 for TLCS +AT-II vs. TLCS alone.(**g**) Acridine orange staining. Treatment with 50 µmol/L of GCDCA induces nuclear condensation and fragmentation (white arrows) which is blocked with 100 nmol/L AT-II at 15 hours-time point.

TNFα (20 ng/ml)/ActD (200 ng/ml), menadione (at 50 µM) and GCDCA (at 50 µM) induce caspase-3 activity in primary rat hepatocytes peaking after 16 hours-, 9 h- and 4 h-exposure, respectively [27], [29]. The effect of AT-II on cytokine-, oxidative stress- and bile acid-induced caspase-3 activity in primary hepatocytes was investigated at these time points. Exposure of primary rat hepatocytes to AT-II (100 nmol/L) alone did not induce caspase-3 activity (Fig. 1a), nor did it reduce the caspase-3 activity in TNFα/ActD-treated or menadione-treated rat hepatocytes (Fig. 1a and 1b). In contrast, AT-II significantly inhibited the GCDCA-induced caspase-3 activity (Fig. 1c; −50% approximately, p<0.05), which was concentration-dependent (Fig. 1d ). In all subsequent experiments a concentration of 10^−7 ^mol/L (100 nmol/L) of AT-II was used. AT-II attenuated the GCDCA-induced caspase-3 activity at different apoptosis-inducing concentrations of GCDCA (50–200 µM; Fig. 1e). At higher GCDCA concentrations, the primary mode of cell death shifts to necrosis [28], which is not prevented by AT-II (Fig. 1e). To determine whether AT-II is also protective towards cytotoxic effects of other bile salts, the effect on tauro-lithocholic acid-3 sulfate (TLCS)-induced caspase-3 activity was studied. Similar as for GCDCA, AT-II reduced TLCS-induced caspase-3 activity in primary rat hepatocytes with approximately 50% (Fig. 1f).

AT-II may delay, rather than prevent GCDCA-induced apoptosis in rat hepatocytes. To establish true protection by AT-II, GCDCA-treated hepatocytes were stained with acridine orange after 6 and 15 h exposure with and without AT-II. Nuclear fragmentation and condensation, markers for end-stage apoptosis, became detectable 6 hours after the addition of GCDCA and increased over time (shown for 15 h treatment; Fig. 1g). Nuclear fragmentation and condensation were reversed to control levels when GCDCA-exposed hepatocytes (after 15 h) were co-treated with AT-II (Fig. 1g). Importantly, AT-II did not induce necrosis in hepatocytes, nor did it increase the number of necrotic cells after exposure to GCDCA (data are not shown.).

### The Protective Effect of Angiotensin II is Mediated via the Angiotensin Receptor Type -1

**Figure 2:**
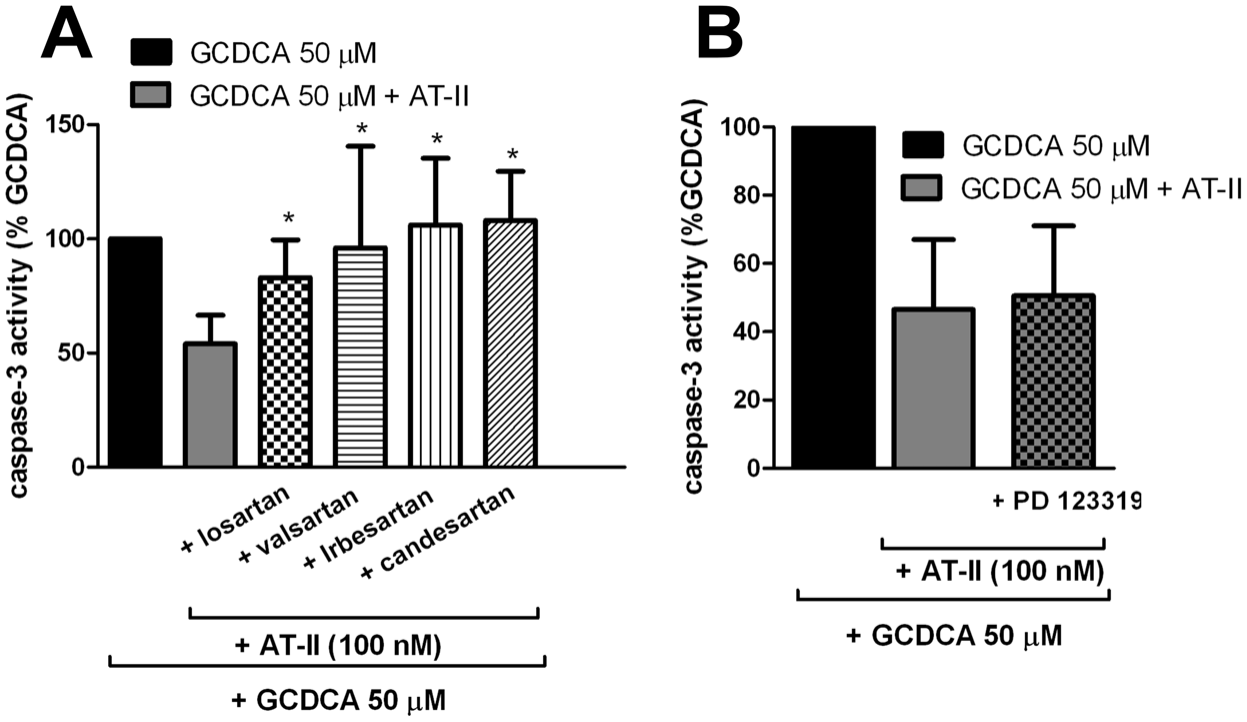
The protective effect of angiotensin II (AT-II) is mediated via angiotensin II type-1 receptor (AT-1R). (**a**) Hepatocytes were treated with 1 µmol/L of selective AT-1R antagonists (Losartan, Valsartan, Irbesartan and Candesartan) 30 minutes prior to the addition of GCDCA (50 µM)+AT-II (100 nmol/L). One-way ANOVA test was used to determine the significance of differences between experimental groups (N = 5). *P<0.05 for GCDCA+ AT-II+ AT-1R antagonists vs. GCDCA+ AT-II. (**b**) Hepatocytes were treated with 1 µmol/L of the selective AT-2R antagonist (PD 123319) 30 minutes prior to the addition of GCDCA (50 µM)+AT-II (100 nmol/L).

AT-II signaling depends on the presence of AT-II receptors in cellular membranes. AT-1R but not AT-2R has previously been shown to be expressed in rat hepatocytes [3]. We also detected significant mRNA expression for AT-1R in rat hepatocytes comparable to those in activated rat hepatic stellate cells, used as positive control (data are not shown). The role of AT-1R and AT-2R in the protective effect of AT-II against GCDCA-induced apoptosis was investigated using specific antagonists of AT-1R (various sartans) or AT-2R (PD123319). The protective effect of AT-II against GCDCA-induced apoptosis was completely abolished in the presence of the various sartans tested (losartan, valsartan, irbesartan and candesartan), strongly indicating that the protective effect of AT-II is mediated through AT-1R (Fig. 2a). In contrast, inhibition of AT-2R with PD123319 did not affect the protective action of AT-II against GCDCA-induced apoptosis in hepatocytes (Fig. 2b).

### Time Window of Anti-apoptotic Action of Angiotensin II

**Figure 3:**
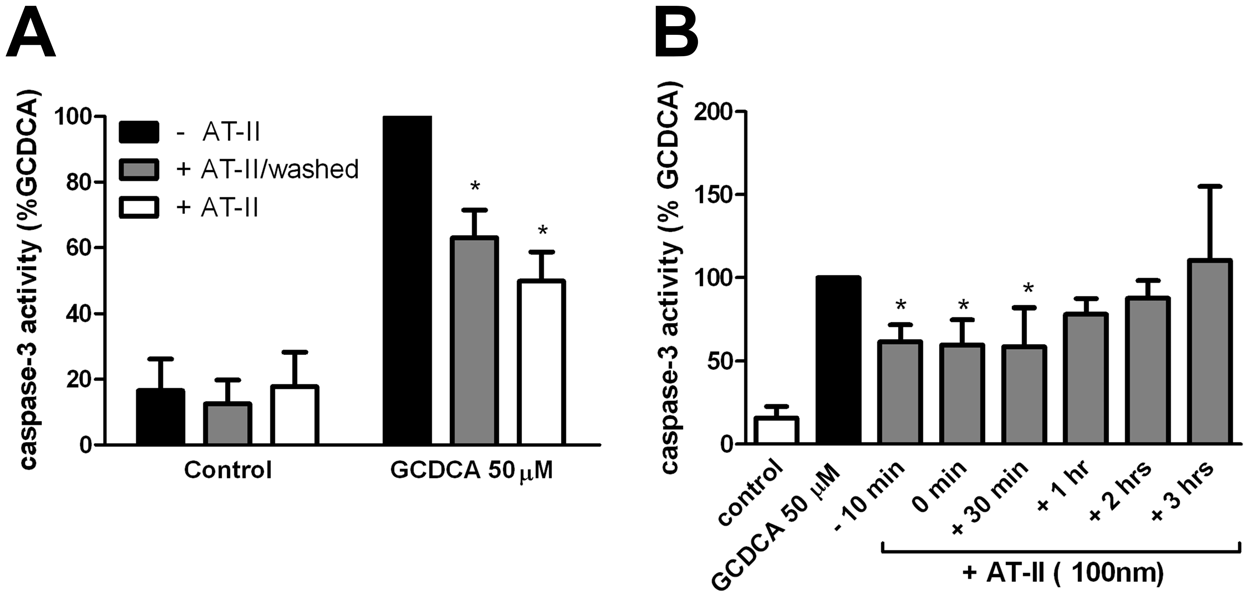
The protective effects of angiotensin II (AT-II) are induced rapidly. (**a**) Hepatocytes were pre-incubated with 100 nmol/L of AT-II for 10 minutes after which cells were washed and exposed to 50 µmol/L of GCDCA alone (+ AT-II/washed) or with simultaneous addition of AT-II (+ AT-II). Two-way ANOVA test was used to determine the significance of differences between experimental groups (N = 4). * P<0.05 for GCDCA+AT-II/washed vs. GCDCA 50 µM and for GCDCA+AT-II vs. GCDCA 50 µM. (**b**) hepatocytes were stimulated with 50 µmol/L GCDCA for 4 hours (GCDCA 50). AT-II (100 nmol/L) was added 10 minutes prior to (−10 min), simultaneous with (0 min) or 30 minutes (+30 min), 1 hour (+1 hr), 2 hours (+2 hrs), 3 hours (+3hrs) after the addition of GCDCA. One-way ANOVA test was used to determine the significance of differences between experimental groups (N = 4). *P<0.05 for −10 min of AT-II, 0 min of AT-II, +30 min of AT-II vs GCDCA alone.

We next investigated whether AT-II needs to be present during the GCDCA treatment to protect hepatocytes against apoptosis. Hepatocytes were pre-incubated with AT-II for 10 minutes. Medium was removed and cells washed and then exposed to GCDCA in fresh medium with or without AT-II for 4 hours. Caspase-3 activity was inhibited in the presence of AT-II and the protective effect of AT-II persisted in hepatocytes exposed to GCDCA in fresh medium without AT-II (Fig. 3a). Furthermore, addition of AT-II up to 30 minutes after the start of the GCDCA treatment still exerted maximum protection against GCDCA-induced apoptosis (Fig. 3b). No protective effect of AT-II was detected when AT-II was added at later time points (1–3 h after GCDCA treatment; Fig. 3b). These data show that the anti-apoptotic actions of AT-II are rapidly induced and suggest the involvement of protein kinase signal transduction pathways in the protective actions of AT-II against GCDCA-induced apoptosis.

### Anti-apoptotic Action of Angiotensin II Depends on the Combined Activation of ERK1/2, p38 MAP Kinase, PI3kinase and Protein Kinase C

**Figure 4:**
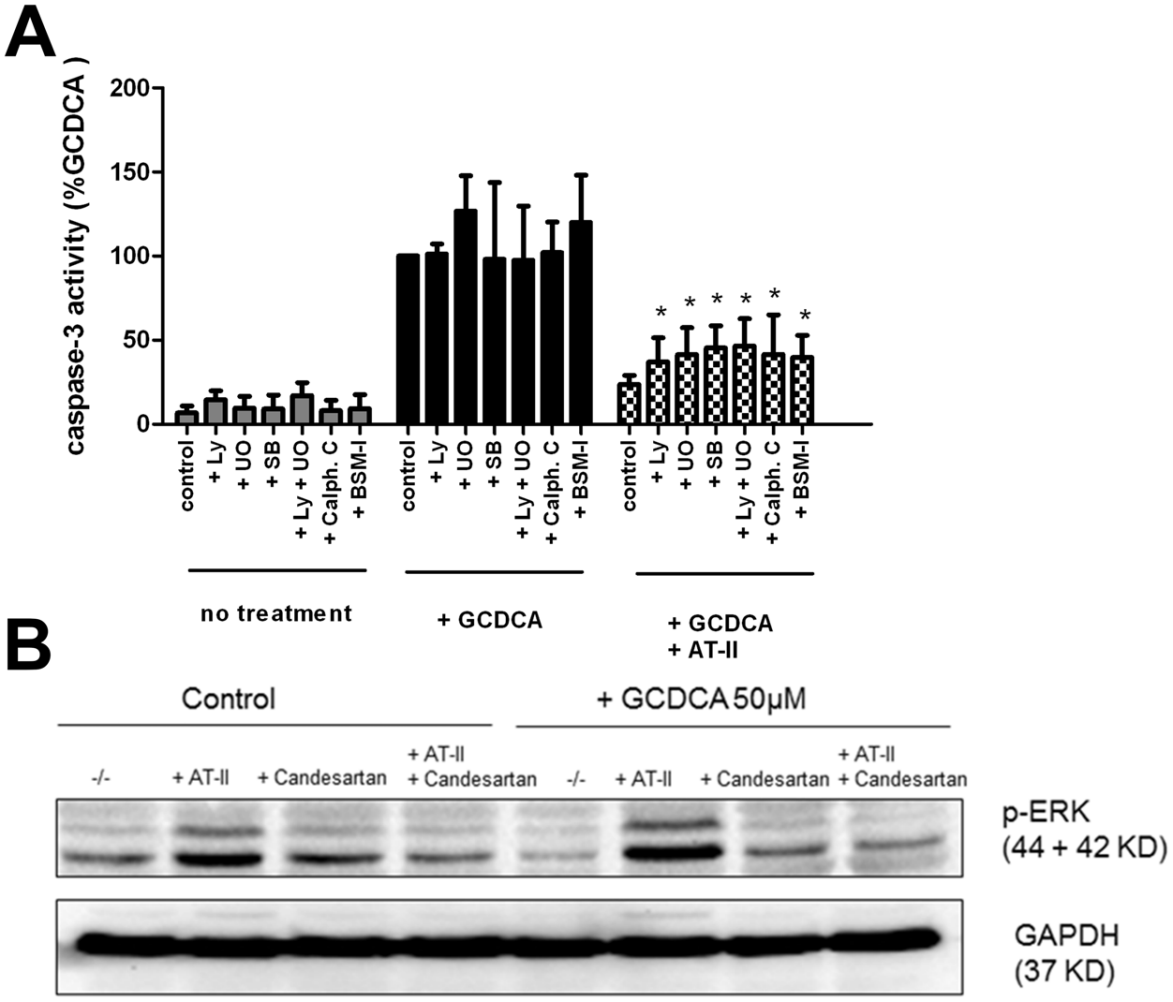
The protective effect of angiotensin II (AT-II) is dependent on the activation of specific kinases. (**a**) Caspase-3 activity in rat hepatocytes treated with 50 µmol/L of GCDCA in the presence and absence of 100 nmol/L AT-II and with or without the inhibitors of ERK MAPK (10 µmol/L of U0126; U0), p38 MAPK (10 µmol/L of SB 203580; SB), PI3K (50 µmol/L of LY 294002; LY) and PKC (1 µmol/L of Calph. C or BSM-I). Unpaired one-tailed student t-test was used to determine the significance of differences between experimental groups (N = 4). *P<0.05 for GCDCA+AT-II+ LY vs. GCDCA+ AT-II, for GCDCA+AT-II+ SB vs. GCDCA+ AT-II, for GCDCA+ AT-II+U0 vs. GCDCA+ AT-II, for GCDCA+ AT-II+U0+LY vs. GCDCA+ AT-II, for GCDCA+ AT-II+Calph.C vs. GCDCA+AT-II and for GCDCA+AT-II+BSM-I vs. GCDCA+AT-II. (**b**) Western blot analysis for phosphorylated ERK1/2 MAPK in cell lysates of GCDCA-exposed hepatocytes (10 min) with and without AT-II. ERK1/2 MAPK phosphorylation was inhibited in the presence of AT-1 antagonist (1 µmol/L of candesartan).

To investigate whether specific kinase pathways are involved in the anti-apoptotic effects of AT-II, GCDCA-exposed rat hepatocytes were co-treated with inhibitors of MAPK, PI3K and PKC. These inhibitors alone do not induce caspase-3 activity in rat hepatocytes, nor do they enhance the GCDCA-induced caspase-3 activation. (Fig. 4a). The protective effect of AT-II against GCDCA-induced apoptosis was significantly, albeit partially, abolished by inhibition of ERK1/2, p38 MAP kinases and PI3 kinase pathways (Fig. 4a). Additive effects were observed when ERK1/2 and p38 MAK kinase were inhibited simultaneously. In support, AT-II increased the levels of phosphorylated ERK1/2 MAPK in control hepatocytes as well as in the presence of GCDCA, which was abolished by the AT-1R antagonist candesartan (Fig. 4b). In the presence of PKC inhibitors, the protective effect of angiotensin II against GCDCA-induced caspase-3 activation was also abolished significantly (Fig. 4a).

### Angiotensin II Attenuates GCDCA-induced Endoplasmic Reticulum Stress

**Figure 5:**
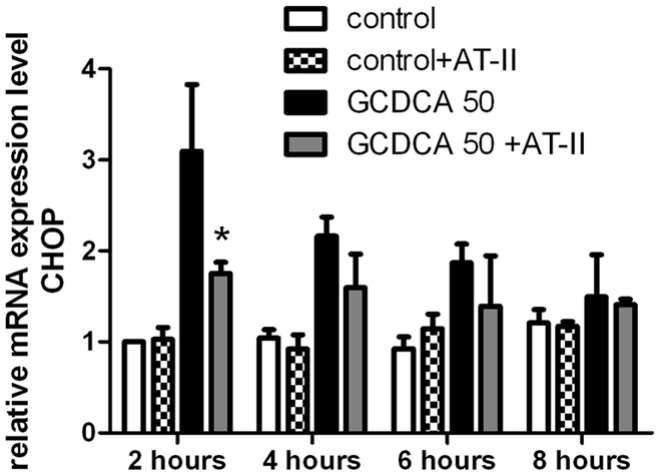
Angiotensin II (AT-II) attenuates GCDCA-induced ER stress in rat hepatocytes. (**A**) QPCR analysis of Chop mRNA (ER stress marker) in rat hepatocytes treated with 50 µmol/L GCDCA at several time points with or without 100 nmol/L of AT-II using cDNA of hepatocytes. mRNA expression level is presented relative to control. One-way ANOVA test was used to determine the significance of differences between experimental groups (N = 3).* P<0.05 for GCDCA +AT-II vs. GCDCA alone at 2 hours.

Besides kinase signaling pathways, hydrophobic bile salts, such as GCDCA, may also induce apoptosis by increasing ER stress and induction of the unfolded protein response via transcription factors like CHOP [30], [31]. Induction of CHOP is a sensitive marker for ER stress and CHOP-knockout mice are protected against cholestasis-induced hepatocyte apoptosis [32], [33]. Indeed, GCDCA treatment of rat hepatocytes lead to a transient increase in CHOP mRNA levels that peaked at 2 hours after GCDCA treatment, after which they gradually decrease to control levels after 4–8 hours (Fig. 5). AT-II prevented the GCDCA-induced expression of CHOP in rat hepatocytes at all different time points tested, while it did not exert any effect on CHOP levels in control hepatocytes (Fig. 5).

## Discussion

In this study, we report that angiotensin II (AT-II) protects primary rat hepatocytes specifically against bile salt-induced apoptosis. We demonstrate that the protective effects of AT-II are mediated via AT-1R signal transduction and activation of ERK, p38 MAPK, PI3K and PKC signaling pathways. In addition, we show that AT-II treatment reduces the bile salt-induced expression of CHOP, an ER stress-induced effector peptide, in primary rat hepatocytes. These findings suggest that inhibition of systemic and/or tissue RAS pathway in (cholestatic) liver fibrosis should be carefully monitored as it blocks the hepatoprotective action of AT-II on liver parenchymal cells.

AT-II antagonists (ACEi and/or ARB) are considered as therapeutics for fibrotic liver diseases as they prevent HSC activation and proliferation [5]. However, the effect of AT-II antagonist therapy on hepatocytes has not previously been studied. Hepatocytes are the most abundant cell type in the liver. They highly express AT-1R and exhibit numerous responses to AT-II, including the activation of gene transcription [3], [34]–[36]. Therefore, it is likely that AT-II harbors specific roles in (diseased) hepatocytes and AT-II antagonist therapy may lead to hepatotoxicity. Indeed, ACEi- and ARB-induced hepatocellular injury and liver damage has been reported previously [15]–[20]. Several mechanisms have been suggested for ARB-induced hepatotoxicity and cholestasis such as metabolic idiosyncrasy and immune mechanisms. However, the hypothesis that AT-II has hepatoprotective effects and ARBs sensitize hepatocytes to toxic stimuli has not been investigated before. In the present study, we have used several apoptotic stimuli (cytokines, ROS and bile salts) to investigate the effect of AT-II on hepatocyte injury. These apoptotic stimuli boost in (chronic) liver diseases and cause hepatocyte injury. Our data strongly demonstrate that AT-II has an AT-1R-mediated hepatoprotective effect on hepatocytes, which is prevented by several ARBs. It is known that hepatocytes express AT-1R and the up-regulation of this receptor in cholestatic liver injury has been reported, whereas the AT-2R is suggested to be absent or very low expressed in hepatocytes (10, 43). Our data confirms that AT-2R-mediated signaling does not play a major role in hepatocytes cell death or survival (likely due to very low expression or absence of this receptor), however we have not measured the expression levels of AT-2R in our experiment.

The specific hepatoprotective effect of AT-II against bile salt-induced apoptosis but not TNFα- or menadione-induced apoptosis is remarkable. This could be due to the induction of different apoptotic signaling pathways in response to these apoptotic stimuli. Menadione induces apoptosis in hepatocytes via production of reactive oxygen species (ROS) leading to oxidative stress in hepatocytes [29]. The role played by ROS in bile salt-induced cell death in hepatocytes is controversial. Previously, we have shown that ROS are not essential for bile salt-induced cell death in hepatocytes and anti-oxidant therapy does not protect hepatocytes against bile salt-induced apoptosis [28], indicating different mechanisms for bile salt and ROS-induced hepatocyte apoptosis. Interestingly, it has been shown that bile salt-induced ROS generation from mitochondria can inactivate protein tyrosine phosphatase (PTPase) activity resulting in activation of growth factor tyrosine kinase receptors and downstream survival signaling pathways in hepatocytes [37], suggesting that ROS production can have both anti- and pro-apoptotic effects in cells. It has been shown that depletion of mitochondrial DNA reduces the ability of Rho cells to produce ROS in response to certain bile salts and consequently these cells were less sensitive to bile salt-induced toxicity [38]. The results of this study support the concept that mitochondria play a key role in bile salt-induced apoptosis. However, the mitochondrial-mediated apoptotic pathways are not limited to ROS-production. Release of the apoptotic factors such as cytochrome *c,* Apoptosis-Inducing factor (AIF) and endonuclease G can also lead to the activation of effector caspases and apoptosis [39]. TNFα-induced apoptosis in hepatocytes is shown to be mediated via death-receptor-dependent signaling (TNF receptor type 1, TNF-R1), leading to recruitment of Fas associated death domain (FADD), activation of caspase-8 and pro-apoptotic proteins, Bak and Bid [40], [41]. In contrast, it has been shown that bile salt-induced caspase-8 activation is FADD-independent and inhibition of caspase-8 has no effect on bile salt- induced caspase-3 activation [41]. In addition, the crucial role of caspase-6 and caspase-9 activation in bile salt- but not TNF-or etopside-induced activation of caspase-3 in human liver cell lines has been previously described [42]. Although we have not tested the involvement of all these pathways in our study, the observed differences in apoptotic signaling pathways may explain why AT-II-induced signaling pathways cannot counteract the menadione- and TNFα-induced apoptotic signaling, whereas they do protect hepatocytes against bile salt-induced apoptosis.

We provide evidence that the protective effects of AT-II are mediated via a combined activatation of protein kinase signal transduction pathways, as we observed partial effects of specific kinase inhibitors, reversing the protective effects of AT-II. In accordance with our data, previous studies have also indicated that AT-II stimulates activation of ERK, PI3K, p38 MAPK and PKC [43]–[48]. It has been observed that taurine-conjugated UDCA (TUDCA)-induced activation of the ERK, PI3K and p38 MAPK protects primary rat hepatocytes against bile salt-induced apoptosis [27]. Thus, it is likely that AT-II mediated activation of these protein kinases aid to the survival of cholestatic hepatocytes. PKC signaling cascade is suggested to play an important role in AT-II related pathophysiology [45], [49]. Interestingly, PKC-alpha has been reported to inhibit hepatocyte apoptosis through an increase of anti-apoptotic Bcl-2 family proteins such as Bcl-XL [50], [51]. Induction of the expression of anti-apoptotic Bcl-XL because of PKC activation by AT-II may be a part of the hepatoprotective effect of AT-II in cholestatic hepatocytes. However, we have not studied which isoform of PKC in particular mediates the heptoprotective effect of AT-II.

In addition, AT-II attenuated the bile salt-induced CHOP expression. CHOP is one of the most sensitive markers for ER stress that activates the ER stress-induced apoptotic pathway [52]. Overexpression or microinjection of CHOP protein can induce cell cycle arrest and/or apoptosis [53], [54]. CHOP enhances the expression of the proapoptotic BH3-only protein Bim and the cell surface death receptor TRAIL receptor 2 (also known as death-receptor 5, DR5) and inhibits transcription of the anti-apoptotic protein Bcl-2 [55]–[57]. In contrast, CHOP disruption in cells leads to reduced apoptosis in response to ER-stress [58]. Indeed, CHOP deficiency reduces cholestasis-induced hepatocyte apoptosis [33]. Activation of ERK 1/2 MAP kinase and PI3K/Akt pathway were found to attenuate the ER stress-induced apoptosis and reduce CHOP expression, leading to cell survival [59]–[61]. Furthermore, activation of PKC signaling pathway may inhibit the ER stress-induced caspase-12 and caspase-3 activation and therefore inhibit apoptosis [62]. Interestingly, inhibition of the ER-stress response by TUDCA is a hepatoprotective mechanism in liver disease [63]. Our data suggest that activation of these protein kinases in AT-II-treated hepatocytes inhibits the ER stress response, leading to hepatocyte survival.

The hepatoprotective effect of AT-II against bile salt-induced apoptosis has great clinical relevance in AT-II antagonist therapy of patients with cholestatic liver fibrosis. As patients with chronic liver diseases usually visit the clinicians when their disease is already advanced and the therapy has to start in already diseased livers containing diseased (e.g. cholestatic) hepatocytes, it is important to clarify the response of hepatocytes to therapy (AT-II vs ARBs). Our data suggest that despite the beneficial effects of ARBs on liver fibrosis, they may hypersensitize the adjacent hepatocytes to certain toxic stimuli such as bile salts, leading to further loss of functional hepatocytes. Therefore, liver function should be closely monitored during the AT-II antagonist therapy of cholestatic liver fibrosis, especially in the early stages of cholestatic liver disease (when there is still considerable amount of functional hepatocytes). In addition, targeting AT-II antagonists specifically to hepatic stellate cells can be a good solution in order to prevent hepatocyte loss during the treatment of liver fibrosis. Indeed, a current study has reported that a selectively HSC-targeted Losartan attenuates advance liver fibrosis more effectively than conventional Losartan [64]. Combined UDCA/ARBs therapy could serve as an alternative option to protect hepatocytes while treating liver fibrosis in cholestatic liver diseases, as UDCA has similar hepatoprotective effects as were observed for AT-II in our study and UDCA is already used in clinic for patients with chronic cholestatic diseases [65]. In summary, we show that AT-II protects primary rat hepatocytes against bile salt-induced apoptosis via the AT-1R-mediated activation of the signaling protein kinases and reduction of the ER stress-induced apoptosis in hepatocytes.

## Supporting Information

Table S1 **Sequences of primers and probes used for quantitative PCR analysis.**
(DOC)
