# Supplementary material for: Angiotensin II Protects Primary Rat Hepatocytes against Bile Salt-Induced Apoptosis
Source: PLoS One. 2012 Dec 26;7(12):e52647. doi: 10.1371/journal.pone.0052647 (PMC3530435; doi:10.1371/journal.pone.0052647)
Supplement: Table S1 — Sequences of primers and probes used for quantitative PCR analysis. (DOC) [file pone.0052647.s001.doc]

Table S 1.Sequences of primers and probes used for quantitative PCR analysis

| 18 S  Rat | Sense  Antisense  Probe | 5’-CGGCTACCACATCCAAGGA- 3’  5’-CCAATTACAGGGCCTCGAAA-3’  5’FAM-CGCGCAAATTACCCACTCCCGA- TAMRA3’ |
| --- | --- | --- |
| Chop  Rat | Sense  Antisense  Probe | 5’-TCCTGTCCTCAGATGAAATTGG- 3’  5’-TCAAGAGTAGTGAAGGTTTTTGATTCT-3’  5’FAM-CACCTATATCTCATCCCCAGGAAACGAAGA- TAMRA3’ |
| AT-1R Rat | Sense  Antisense  Probe | 5’-GCCCAGCGGGACTCTGT- 3’  5’-CTAATGTAGATAATGTCCAGGAAGATGGT-3’  5’FAM-TTGGCATGTTTCTTGGTGGCTTGGT-TAMRA3’ |
